# The effects of declarative learning on early and late motor skill learning

**DOI:** 10.1038/s41539-025-00386-3

**Published:** 2025-12-27

**Authors:** Mohan W. Gupta, Timothy C. Rickard

**Affiliations:** 1 Princeton University, Princeton, NJ USA; 2 University of California San Diego, San Diego, CA USA

**Keywords:** Psychology, Human behaviour

## Abstract

Motor skill learning and performance is driven by the interplay between declarative and nondeclarative systems, which can complement or interfere with one another depending on task demands. In this study, we investigated whether an intervening declarative cued-recall task impairs motor skill performance in a finger-tapping-task and assessed three hypotheses: the consolidation disruption hypothesis, the shared resource hypothesis, and the breakdown of inhibition hypothesis. Intervening declarative tasks placed late in training failed to affect motor performance. In contrast, when introduced early in training (Experiment 3), motor performance appears to have been transiently impaired. Despite this impairment, there was no association between the intervening declarative task performance and motor impairment. We discuss the ramifications of these findings with regard to other motor skill tasks and intervening declarative tasks.

## Introduction

An emerging consensus is that motor skill learning involves at least two systems: declarative and nondeclarative^1–4^. Declarative processes likely dominate initial rapid learning, while slower, nondeclarative processes drive long-term performance gains. While these systems are complementary during motor learning, they may interfere with one another when independent motor and declarative tasks—tasks that assess explicit memory for facts or events (e.g., free recall, cued recall, recognition, source/temporal-order judgments)—are interleaved^5–11^. For example, Brown and Robertson^5^ first trained subjects on a serial response time task in which key presses were made in response to a set of visual cues, yielding a key press sequence of which participants were not made aware. Next, participants either studied and were tested on a word list (invoking declarative memory encoding) or they counted vowels in random character strings (invoking minimal memory declarative encoding). The word list task impaired subsequent performance on the serial response time task more than did the vowel string task (c.f. Brown and Robertson,^11^ for the opposite finding of motor performance facilitation following word list learning).

Any of at least three distinct processes may explain declarative learning impairment on motor performance. Brown and Robertson^5^ suggested that the declarative task disrupts offline motor skill consolidation, reducing the post-rest gain effect—the finding that motor sequence performance improves following short rests (5–30 min). This *consolidation disruption hypothesis* is buttressed by recent findings suggesting that facilitating micro-consolidation occurs on a time scale of seconds during breaks (5–30 seconds), putatively via hippocampal-neocortical replay^12–14^, c.f.^15^. Possibly, declarative learning during the rest period disrupts that neural replay, impairing memory consolidation and resulting in performance impairment.

Reactive inhibition (RI) provides an alternative process by which a declarative task inserted during a rest break may impair motor learning. Empirically, RI refers to the observed worsening of performance as one continually performs a motor task^14–18^. RI dissipates during breaks. Brief breaks can result in only partial dissipation, leading to cumulative accrual of RI across training trials. Longer rest periods, such as five to 15 min, facilitate a more complete dissipation, along with a substantial sequence performance improvement following the rest period^14–18^. It is possible that the effect of an inserted declarative task is not to interfere with micro-consolidation but rather to disrupt the dissipation of RI. At a mechanistic level, RI has been associated with a breakdown in surround inhibition in the somatosensory cortex, a process that normally suppresses extraneous neural activity and reduces antagonistic muscle coactivation^19–21^. This breakdown increases the excitation-inhibition ratio in the somatosensory cortex, broadens neural tuning curves, and results in less efficient and more effortful motor commands. During rest periods, inhibitory control in the somatosensory cortex is thought to recover, restoring balance and alleviating the effects of RI. Declarative tasks performed during these recovery intervals may disrupt this restoration process (the *breakdown of inhibition hypothesis*). By competing for shared neural and cognitive resources^7^, declarative tasks could delay or impair the re-establishment of inhibitory control in somatosensory cortex, thereby exacerbating the excitation-inhibition imbalance. Thus, declarative tasks may disrupt the dissipation of RI and reduce post-rest performance gains.

A third candidate process is resource competition between declarative and motor tasks (the *resource hypothesis*). In a visuomotor adaptation task, Keisler and Shadmehr^7^ suggested that declarative and motor learning share a limited cognitive resource, in particular a fast learning process that is linked to declarative learning^1–3^. An intervening declarative task competes for this shared resource, disrupting the processing of the motor memory and diminishing performance gains. Although that shared resource has not been further specified since, we will make an extension here. The limited cognitive resource could be conceptualized as the finite capacity for declarative information processing. This mirrors evidence suggesting early motor sequence learning gains are largely driven by an increase in the planning horizon—the number of future motor elements one can prepare ahead of execution^22,23^. Thus, it may be the case that online planning capacities are fundamentally constrained by the number of discrete elements that can concurrently enter the fast learning process.

**Fig. 1:**
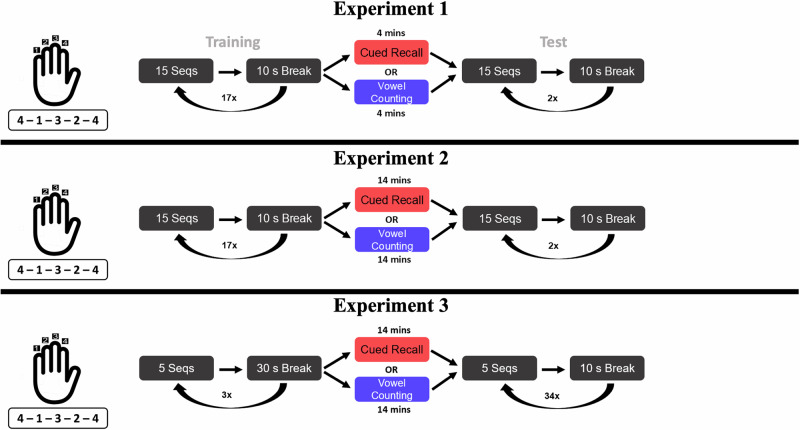
Experimental methods. Participants learned to perform a sequence (4–1–3–2–4) by repeatedly typing the sequence. After some training, participants either did an intervening cued-recall task or a vowel counting task. After performing that task for a set number of trials, with time equated between the tasks, they returned to performing the sequence. In the first two experiments, the intervening tasks were placed late in training, whereas in the third experiment it was placed early in training.

The consolidation-disruption hypothesis suggests that a declarative task commandeers hippocampal–neocortical micro-consolidation during any break period, so it should reduce the post-rest performance gain regardless of the amount of prior motor training. In contrast, the breakdown-of-inhibition hypothesis traces that behavioral impairment to an RI-recovery failure: by impairing the rebound of surround inhibition in the somatosensory cortex, the declarative task reduces the degree to which RI is dissipated during rest. Therefore, its impairment scales with the amount of cumulative RI accrual during training, prior to the rest period. Finally, the resource-competition hypothesis limits impairment to *early learning*, when a fast, declarative-linked process may dominate skill acquisition. To disentangle these three competing hypotheses, we designed a trio of finger-tapping task (FTT) experiments (Fig. 1)^15^. All experiments involved the same FFT and intervening word list and vowel counting tasks. The Experiments differed with respect to four factors: the number of sequences per trial, the duration of the break between trials, the number of training and test trials, and the duration of the intervening tasks.

## Results

### Experiment 1

To ensure that errors did not compromise our RT interpretations, we tested for differences in error rates between the two groups. The error rate was calculated for each participant as the number of incorrect key presses prior to each correct sequence within each trial. Averaging over sequences, trials, and participants in the training phase, the error rate was 0.59 and 0.47, key presses per sequence in the cued-recall and vowel groups, respectively. A two-sample t-test on the participant-level error rate revealed a non-significant group effect, *t*(96) = 1.08, *p* = 0.28, *d* = 0.15, *BF*_*01*_ = 2.81. The mean error rates on the two test trials were 0.31 and 0.47 key presses in the declarative and vowel groups, respectively, *t*(96) = −0.651, *p* = 0.51, *d* = −0.093, *BF*_*01*_ = 3.89.

**Fig. 2:**
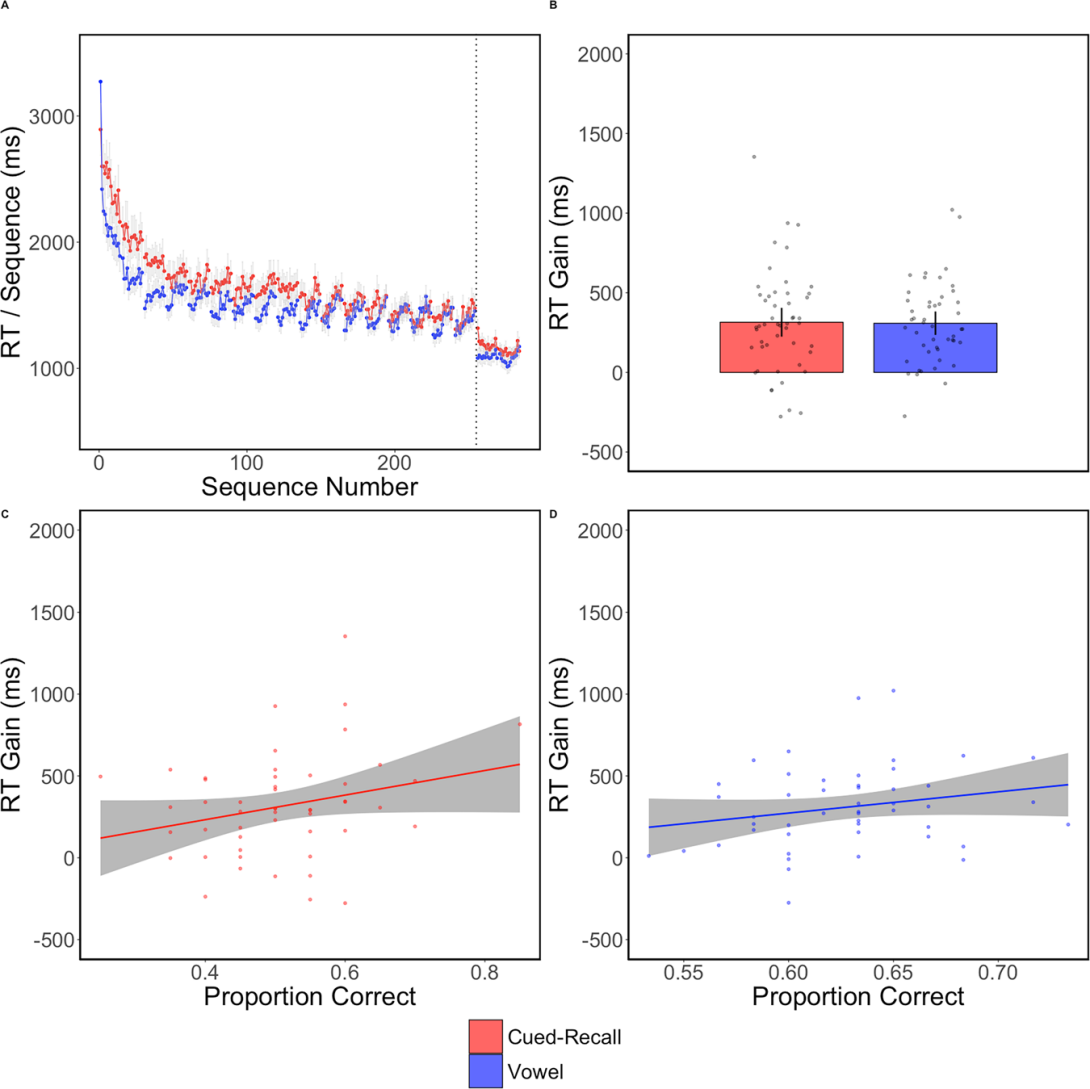
Experiment 1—No declarative impairment effect in late learning. **A** In each panel, red refers to the cued-recall group and blue refers to the vowel group. Each dot is the average RT of one correctly completed sequence. The dots connected by a line indicate they are part of the same trial. Both groups had the same number of sequences per trial and equal break time. The first sequence of each trial was considered a warm-up sequence and removed from analyses and plotting. The gray error lines are sequence-level standard errors. **B** The bar plot shows the final test mean RTs with each dot indicating an individual participant. Error bars are 95% confidence intervals. **C** An Ordinal least-squares regression indicated that there was no relationship between the RT Gain and the proportion correct on either the cued-recall task (**D**) or the vowel counting task.

Strong trial-level RI effects (i.e., progressively larger sequence RTs across sequences within trial) are visually evident in Fig. 2A, starting at about trial 3. To confirm that effect statistically, we performed a two-sample t-test on the RT difference between the last sequence and the second sequence (the first sequence having been removed as warm-up), averaging across the last two training trials. This minimized the influence of learning rate on our measurement. In the vowel group, *t*(47) = −3.29, *p* = 0.0019, *d* = −0.48, *BF*_*10*_ = 16.37. In the cued-recall group, *t*(49) = −2.44, *p* = 0.018, *d* = −0.35, *BF*_10_ = 2.25.

To test for performance differences between groups before the intervention, we performed a two-samples t-test on the average RT over the last two training trials and found no significant group difference, *t*(96) = 089, *p* = 0.37, *d* = 0.13, *BF*_*01*_ = 3.3 (Fig. 1A).

The key test for determining whether declarative learning impaired motor sequence learning is on the group difference in the post-rest gain. Based on the mean RT difference between the last two training trials and the two test trials, we found no significant effect: *t*(96) = 0.1, *p* = 0.91, *d* = 0.015, *BF*_*01*_ = 4.68 (Fig. 1B).

The proportion correct on the cued-recall task was 0.51, and on the vowel counting task was 0.63. We found no significant association between post-rest RT gain and proportion correct on either the cued-recall task *F*(1, 48) = 3.37, *p* = 0.07, *r*^*2*^ = 0.046 (Fig. 1C), or the vowel counting task, *F*(1, 46) = 2.38, *p* = 0.13, *r*^*2*^ = 0.028 (Fig. 1D).

In an explicit FTT, we induced a large amount of RI during training by having participants perform 15 sequences during each trial with short breaks between. After training, participants either performed a cued-recall task with word pairs or a vowel counting task. A post-rest gain on the test was observed for both groups. Based on prior findings^6,8–10^, we expected to find a decreased post-rest gain in the cued-recall group relative to the vowel group. However, we observed no significant difference. Further, we found no association between the post-rest gain and intervening task performance for either the vowel counting or cued-recall tasks. While there was a trending association between cued-recall proportion correct and the post-rest gain, it was in the opposite of the expected direction. We further discuss the interpretation of the lack association in the Discussion.

Although our experiment was well powered relative to the extant studies, it is possible that our declarative interference manipulation was weak. For example, in Brown and Robertson^5^^,11^ participants were presented with the 16 item word-lists five times, totaling 80 presentations, and asked to recall the word lists five times, before testing again on the serial response timing task. In this first experiment we only had 20 items presented at a singular time with one chance for recall. Thus, it is possible that we did not generate enough declarative interference with our design. In Experiment 2, we increased the number of items in the vowel counting and cued-recall tasks.

### Experiment 2

Again, we investigated if errors had any influence over our interpretations of our results. Averaging over sequences, trials, and participants in the training phase, the error rate was 0.69 and 0.49 in the declarative and vowel groups, respectively. A two-sample t-test on the mean error rate revealed no significant effect between groups, *t*(103) = 1.24, *p* = 0.21, *d* = 0.17, *BF*_*01*_ = 2.43. On the test trials the mean error rate across trials was 0.51 and 0.58 key presses in the declarative and vowel groups, respectively. A two-sample t-test again yielded no significant group effect, *t*(103) = −0.3, *p* = 0.76, *d* = −0.041, *BF*_*01*_ = 4.66.

**Fig. 3:**
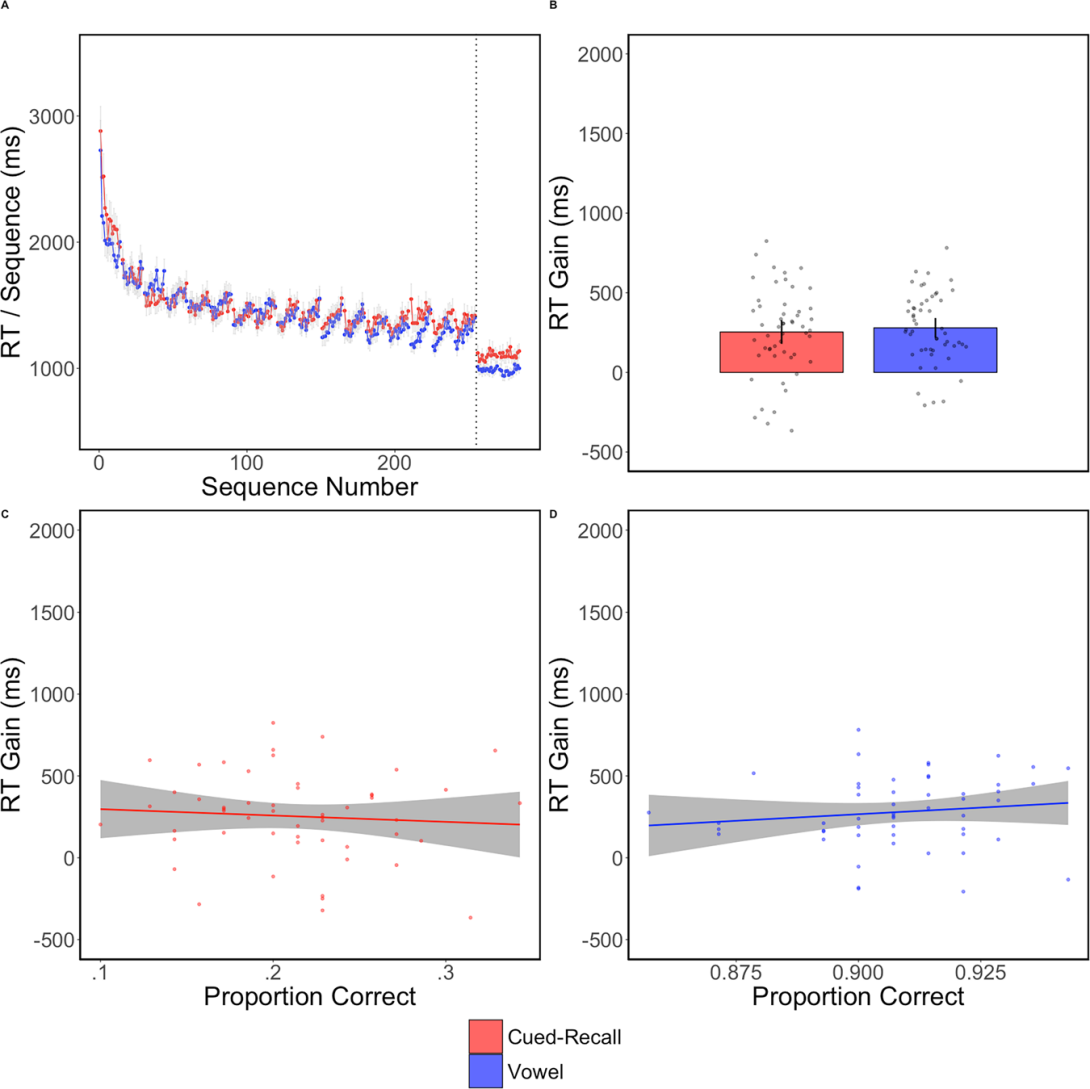
Experiment 2—No declarative impairment effect in late learning. **A** In each panel, red refers to the cued-recall group and blue refers to the vowel group. Each dot is the average RT of one correctly completed sequence. The dots connected by a line indicate they are part of the same trial. Both groups had the same number of sequences per trial and equal break time. The first sequence of each trial was considered a warm-up sequence and removed from analyses and plotting. The gray error lines are sequence-level standard errors. **B** The bar plot shows the final test mean RTs with each dot indicating an individual participant. Error bars are 95% confidence intervals. **C** An Ordinal least-squares regression indicated that there was no relationship between the RT Gain and the proportion correct on either the cued-recall task (**D**) or the vowel counting task.

Again, strong trial-level RI effects are visually present in Fig. 3A starting around trial 3. To minimize the influence of the learning rate on our ability to detect RI, we calculated the RI effect on the last two training trials as we did in Experiment 1. In the vowel group, *t*(51) = −5.35, *p* < 0.0001, *d* = −0.74, *BF*_10_ = 8118.5. In the cued-recall group, *t*(52) = −0.073, *p* = 0.94, *d* = −0.01, *BF*_01_ = 6.66. Further, we found no RT differences between groups averaged over the last two training trials, *t*(103) = 1.5, *p* = 0.14, *d* = 0.21, *BF*_*01*_ = 1.78.

Taking the RT difference between the last two trials of training and the two test trials, we found no significant difference between groups, *t*(103) = −0.55, *p* = 0.59, *d* = −0.075, *BF*_01_ = 4.24.

The proportion correct on cued-recall task was 0.21 and for the vowel counting task was 0.91. We found no associations between post-rest RT gain and task performance in the cued-recall group, *F*(1, 51) = 0.3, *p* = 0.59, *r*^2^ = −0.0058, nor the vowel counting group; *F*(1, 50) = 0.87, *p* = 0.35, *r*^2^ = 0.017.

In Experiment 2, we increased the number of items in the cued-recall task to enhance the potential potency for declarative impairment, relative to Experiment 1. Regardless, we still found no difference in the post-rest gain between the declarative and vowel groups. These results contradict the predictions of both the consolidation and breakdown of inhibition hypotheses, which posit that declarative tasks would impair performance regardless of when they occur during training. Thus, we conclude that neither RI dissipation nor micro-consolidation processes were influenced by the intervening declarative task.

Although there was a significant group difference in final test motor skill performance in the two groups in this experiment, that difference is not the key comparison. Rather, the critical test is for a group difference in the post-rest RT gain, calculated as the difference between end-of-training and test trials. That test mitigates the influence of sampling error in motor ability^24^. This is particularly important in motor skill tasks, since there are substantial variations in human motor ability and performance^25,26^, even among college aged students. Critically, neither experiment had group differences in the post-rest gain, nor associations between post-rest gains and the performance in the declarative or vowel tasks, supporting the absence of an effect of the intervening declarative task on motor learning.

Possibly, those null declarative interference results reflect a dissociation between the fast declarative process, which operates early in training, and the slow nondeclarative process, which drives performance improvements later in training, as suggested by the cognitive resource hypothesis. By the end of training in Experiments 1 and 2, performance gains were likely dominated by nondeclarative processes, making it unlikely under the cognitive resource hypothesis, that a subsequent declarative task would disrupt an unactive. Hence, these results do not rule out the cognitive resource hypothesis, which predicts impairment primarily during early training when the fast process is most active.

To test whether declarative learning selectively affects the early, fast learning process, we designed Experiment 3 with two key modifications: reducing RI to minimize the noise it adds to the signal of underlying motor skill learning and moving the declarative task earlier in training, where the hypothesized fast process should be more active.

### Experiment 3

Errors again did not differ significantly in the two groups. Averaging over sequences, trials, and participants in the training phase (first three trials), the error rate was 0.56 and 0.46 key presses in the declarative and vowel groups, respectively. A two-sample t-test on the error rate revealed no significant effects between groups, *t*(103) = 0.33, *p* = 0.74, *d* = 0.046, *BF*_01_ = 4.61. On the first three test trials, the error rate was 0.52 and 0.41 key presses in the declarative and vowel groups, respectively. A two-sample t-test revealed no significant effect between groups, *t*(103) = 0.49, *p* = 0.63, *d* = 0.067, *BF*_01_ = 4.36.

**Fig. 4:**
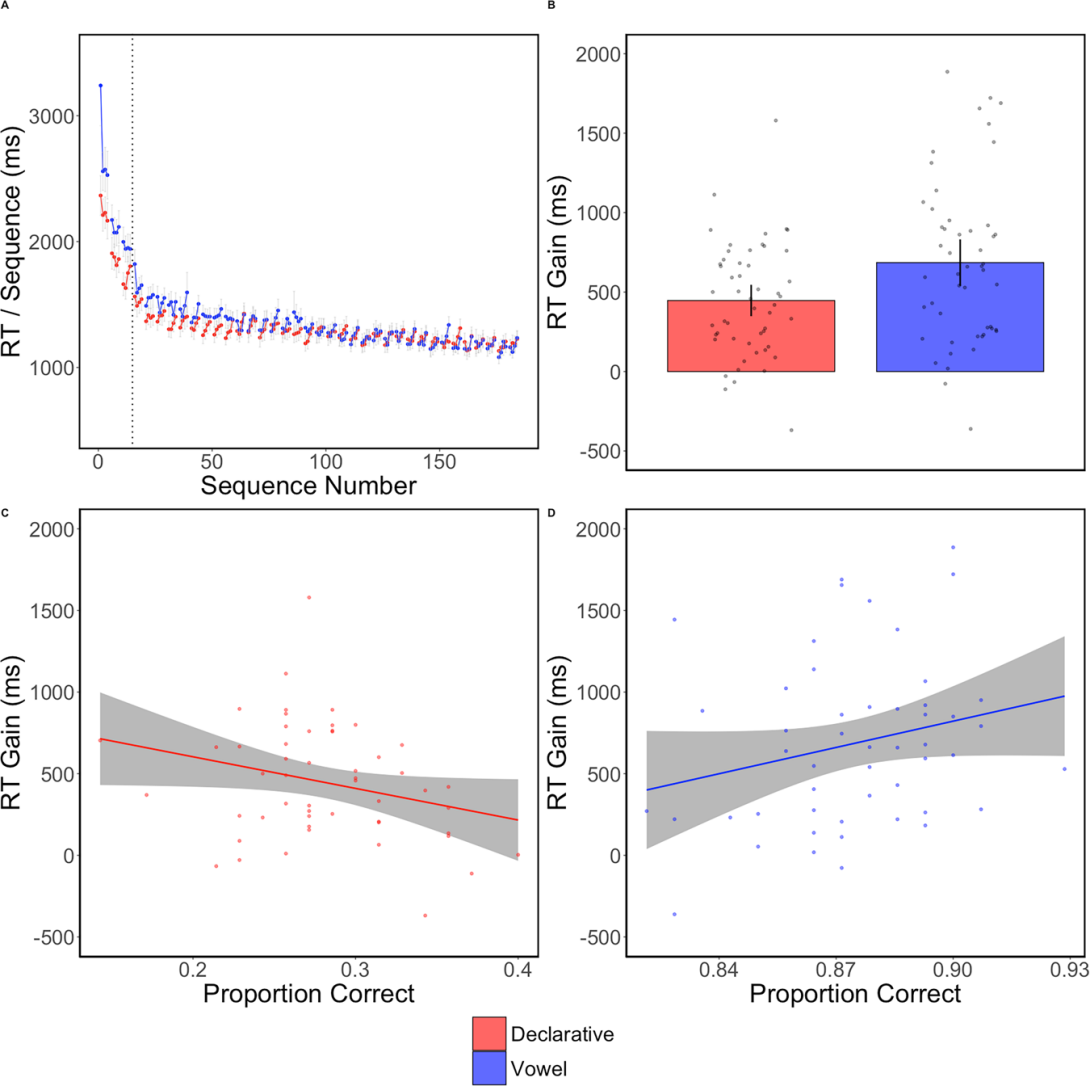
Experiment 3—Declarative impairment effect in early learning. **A** In each panel, red refers to the cued-recall group and blue refers to the vowel group. Each dot is the average RT of one correctly completed sequence. The dots connected by a line indicate they are part of the same trial. Both groups had the same number of sequences per trial and equal break time. The first sequence of each trial was considered a warm-up sequence and removed from analyses and plotting. The gray error lines are sequence-level standard errors. **B** The bar plot shows the final test mean RTs with each dot indicating an individual participant. Error bars are 95% confidence intervals. **C** An Ordinal least-squares regression indicated that there was no relationship between the RT Gain and the proportion correct on either the cued-recall task (**D**) or the vowel counting task.

We found a significant difference between groups averaged RTs over the three training trials (Fig. 4A), *t*(103) = −2.15, *p* = 0.03, *d* = -0.3, *BF*_10_ = 1.59. This effect is likely due to sampling variability of learning rates and initial motor ability since participants were randomly assigned to each group and sampled from the same university student pool. Also likely reflecting sampling variability, we found an insignificant RI effect for the cued-recall group, *t*(52) = 0.73, *p* = 0.47, *d* = 0.1, *BF*_*01*_ = 5.19, and a significant effect for the vowel group in the opposite direction that RI would be in, *t*(51) = 2.49, *p* = 0.016, *d* = 0.35, *BF*_10_ = 2.44. This lack of an effect and effect in the opposite direction is likely driven by the fact that we are measuring RTs early in learning and thus the learning rate would mask or reverse any such RI effects as we observe here.

We found a significant group difference in the post-rest gain, based on the difference between the three training trials and the first three test trials, *t*(103) = −2.06, *p* = 0.042, *d* = −0.28. The Bayes Factor test, however, indicated weak evidence for the alternative hypothesis, BF_*10*_ = 1.33. To investigate if there were any longer-term effects of the declarative impairment, compared the mean sequence RT on the last three post-test trials and found no significant group difference, *t*(103) = 0.43, *p* = 0.67, *d* = 0.059, *BF*_*01*_ = 4.47.

The proportion correct for cued-recall is 0.28 and for the vowel counting is 0.88. We found no significant associations between post-rest RT gains and performance on the cued-recall task, *F*(1, 51) = 2.96, *p* = 0.09, *r*^2^ = 0.055, nor the vowel counting task, *F*(1, 50) = 3.67, *p* = 0.06, *r*^2^ = 0.068.

In Experiment 3, we placed the intervening task after the first three trials (15 sequences) to test whether the declarative task selectively disrupts the fast learning process that underlies early motor skill acquisition. The cued-recall group exhibited a significantly smaller post-rest gain compared to the control group, suggesting that the declarative task impaired motor performance early in training. This impairment effect appears to be temporary, however, as both groups reached equivalent performance levels by the end of the post-test trials, with visually similar performance by sequence 90. Possibly, it may be driven by the cued-recall declarative interference which decreases the memory strength of the sequence, reducing retrieval speed and performance. However, sufficient practice compensates for this initial decrease.

Despite the significant group difference in post-rest gains, Experiment 3 does not unambiguously support the interpretation that the declarative task impaired motor performance. If the declarative task causally interferes with early motor learning or performance, there should be a significant and strong negative association between the post-rest gain and performance on the declarative task. In contrast, here is a non-significant association which only accounts for 5.5% of the variance of the post-rest gain. In our view, even if this association is genuine, this would be very weak evidence that the declarative task meaningfully impairs motor skill learning or performance. Furthermore, the Bayes Factor test on the post-rest gain indicated weak support for the alternative hypothesis. While it is conceivable that our design lacked sufficient power to detect a stronger effect, this seems unlikely given the duration of the declarative task (14 min in our study vs. 72 s–14 min in the referenced studies) relative to motor practice (~30 s) and the large sample size (~105 participants vs. 18–75 per experiment in the referenced studies)^5–7,9,10^. Overall, these findings suggest that while the declarative task may impair early motor performance, the evidence for a causal relationship or a real effect is limited.

## Discussion

In three experiments, we investigated whether an intervening declarative task impairs motor skill learning. We found no compelling evidence that it does. In Experiments 1 and 2, we placed the interfering task after a relatively large amount of training and we did not replicate findings favoring declarative impairment in several motor learning paradigms, including visuomotor adaptation^7,10^, motor sequence learning^5,6^, and general motor movements^9^. With respect to the three theoretical hypotheses we considered, we found no support for either the consolidation disruption or the breakdown of inhibition hypothesis, and only tenuous support for the cognitive resource hypothesis in Experiment 3. Both the consolidation disruption and breakdown of inhibition hypotheses predict motor performance impairment regardless of when the intervening declarative task is placed in the course of training. Proponents of the consolidation hypothesis posit that “most, if not all motor learning occurs offline”^13^ and thus a disruption to this consolidation process will impair learning and performance, regardless of whether it occurs late or early in learning. The breakdown of inhibition hypothesis similarly predicts that the declarative task will inhibit the dissipation of RI, resulting in motor performance impairment regardless of whether the intervening declarative task occurred late or early in training. Based on the results of Experiments 1 and 2, we can reject both of those hypotheses, at least for the FTT.

In Experiment 3, when the intervening declarative task was placed early in learning, we indeed observed a motor performance impairment as suggested by the decreased RT gain in the cued-recall group. That result is consistent with the resource hypothesis^7^, according to which the fast process is a shared resource between the declarative and motor tasks. However, there are two issues with that interpretation and with the more general conclusion that a declarative task casually impairs early motor skill learning. Firstly, the Bayes Factor test in Experiment 3 indicates weak evidence for this effect (*BF*_10_ = 1.33). Secondly, we failed to find the expected association between the post-rest gain and accuracy in the declarative task that has been observed in some other studies. While the association is trending (*p* = 0.09), the amount of variance explained by our regression model is minimal (*r*^*2*^ = 0.055). Overall, if there is an impairment effect on early motor skill learning, it appears to be quite minimal, at least in the FTT.

Alternatively, only a minimal threshold of declarative effort may be required to have impairment on motor performance^6^. Thus, there could plausibly be no association between declarative and motor performance, as shown in our results. Interestingly, not all studies report whether there was an association^6,7,9^, making it unclear whether an association always exists. Both Kim^10^ and Brown and Robertson^5^ found significant negative associations. However, Brown and Robertson^11^ found the opposite, facilitating effect of an intervening declarative task on motor performance using a nearly identical design and item set as did Brown and Robertson^5^. These inconsistent associations may be due to a variety of factors such as task difficulty, whether the motor task is implicit or explicit, the use of different motor effectors, or simply sampling error. Further research is needed to understand these discrepancies. Finally, our null results are not without company^27–29^, suggesting that either the effect of declarative impairment on motor performance is non-existent or that the true effect size is much smaller than suggested in most of the literature.

Why then have other studies found significant effects of declarative impairment on motor performance? In a study similar to the current Experiments 1 and 2, Gagné and Cohen^6^ found a significant impact of a declarative task on motor performance. There are three critical differences between our design and theirs: the declarative interference task, the treatment in the control group, and the rest time. Instead of a linguistic based declarative task, they used a visual search task where participants located targets in pictures. Both the control and experimental groups performed this task after training on day one. On the second day, only participants in the experimental group were asked to remember the locations of the targets. In contrast, the control group had no intervening task and was immediately tested on the motor skill task. Possibly, the observed performance difference between the two groups is attributable to greater fatigue in the experimental group from performing an extra task, rather than declarative interference. Finally, circadian rhythm factors may have impacted their results since they did not strictly control task time of day between groups^27,30,31^.c.f^32^.

There are several limitations in the current work. Firstly, the FTT is a relatively simple motor skill. It is learned quite quickly, such that declarative-based motor skill learning is likely to only drive early learning. In contrast, more complex skills, like piano learning, along with some implicit motor sequence tasks, may have more protracted performance gains driven by declarative learning. For example, in piano learning most of the initial learning must be declarative since one has to learn which key corresponds to a particular pitch, which notes are grouped together to make a scale, the proper body position, and fingering positions, etc^33^. Further, piano teachers will often use metaphors or mnemonics to help students learn or perfect a technique^34,35^. This points to a strong and persistent declarative component in more complex motor skill learning.

A study by Xie et al.^9^ points to this possibility. They investigated the memory effects of learning semantically and non-semantically related words after training on different general movements. Compared to a control group, the recall of general movements was facilitated when paired with semantically related words (i.e., “squatting down, leg lifting, waving hands, jumping, running, rope skipping, quick marching, leg pressing, raising arms, expanding chest”) and recall of general movements was worsened when paired with semantically unrelated words (i.e., “fencing, soccer, bunker, swimming, inverted, Kung Fu, weightlifting, cooking, shotting [sic], piano”). In a follow-up experiment, they further confirmed that the enhancement of recall for the semantic association group only occurred if the ordering of words matched the ordering of the movements whereas when the words were unordered, there was no improvement. This suggests that learning the semantically related ordered words was sufficient to reactivate the temporal ordering of the learned movements, whereas the unordered movements were not sufficient. Further, the unrelated words interfered with the recall of the movements, possibly disrupting the storage of the temporal ordering of those movements. Future research should investigate which types of motor tasks can be impaired or facilitated by declarative learning and the degree to which semantic relatedness of the declarative information enhances or inhibits motor performance.

Another issue with interpreting the Experiment 3 results is the numerically higher RTs of the vowel group on the first training trial. This initial difference could lead to faster acceleration in the vowel group toward the apparently shared within-session RT asymptote (at the end of the test trials) for the two groups. That effect could potentially account for the small but statistically significant group difference in the observed post-rest gains, constituting an alternative to the declarative interference account. It is important to note that this effect was observed in a reasonably large sample of young, college-aged students from the same university. This demographic homogeneity should limit sampling variability in motor performance. Future studies investigating early motor skill learning should heed the substantial sampling variability inherent in early motor performance and consider its potential impact on results.

Our results largely argue against any motor performance impairment from an intervening declarative task. In two experiments, an intervening declarative task placed late in learning failed to impair motor performance, while in a third experiment, placing the task earlier in training resulted in temporary motor impairment. Critically, there was no predicted association between motor performance impairment and declarative performance. Further, the RT impairment in Experiment 3 resolved by the end of the post-test trials, suggesting the effect was temporary and perhaps not causally linked to declarative learning. These findings, particularly experiments with manipulations during early learning phases, like Experiment 3, highlight that there is significant motor performance sampling variability which may yield group differences regardless of whether an intervention has an effect.

## Methods

### Statistical analyses

The dependent measure for the FTT was milliseconds to complete a correct sequence (i.e., response time; or RT). The duration of each keypress within a sequence was measured as the time (in milliseconds) between temporally adjacent keypresses. To reduce noise in the data, we log-transformed each keypress duration. The mean of the logged keypress durations was then calculated for each sequence and participant. We then anti-logged those means and multiplied them by five to obtain a measure of sequence RT in milliseconds. The first completed sequence was removed from each trial prior to further analysis due to the consistently longer RTs on those sequences, indicative of warm-up^14,15^. Further, all RT analyses were performed on the correct sequence RTs.

To test our hypotheses, we used both frequentist statistics and Bayes factors. All t-tests were two-tailed with an *a* < 0.05. For Bayes factor tests, the *r* (prior) value was set to 0.707 based on the recommended default Cauchy prior value^36^. We used Raftery’s guidelines to interpret the Bayes factor, where 1–3 is weak, 3–20 is positive, 20–150 is strong, and >150 is very strong evidence for the null or alternative hypothesis^37^. The Bayes factor for the null is labeled BF_01_, whereas the Bayes factor test for the alternative hypothesis is BF_10_.

### Participants

In Experiment 1, ninety-eight self-reported right-handed undergraduate students from the University of California, San Diego were recruited to participate in the study. Forty-eight participants were part of the vowel group (age = 20.15, *F* = 79.2%). Fifty participants were part of the cued-recall group (age = 20.38, *F* = 78%). Informed consent was given via button press. All procedures were approved by the institutional review board of the University of California, San Diego.

In Experiment 2, one-hundred and five self-reported right-handed undergraduate students from the University of California, San Diego were recruited to participate in the study. Fifty-two participants were part of the vowel group (age = 20.42, *F* = 73.1%). Fifty-three participants were part of the cued-recall group (age = 20.83, *F* = 88.7%). All procedures were approved by the institutional review board of the University of California, San Diego.

In Experiment 3, one-hundred and five self-reported right-handed undergraduate students from the University of California, San Diego were recruited to participate in the study. Fifty-three participants were part of the vowel group (age = 20.17, *F* = 80.8%). Fifty-two participants were part of the cued-recall group (age = 20.91, *F* = 90.6%). All procedures were approved by the institutional review board of the University of California, San Diego.

### Materials

We used English word pairs in the cued-recall task, which were weakly associated and drawn from Nelson et al.^21^ Free Association Norms database. We drew a subset of words that have been used in our prior work (e.g. Gupta et al.,^15^ see Supplementary Table 1). Words were between 4 and 7 letters in length. Mean forward associate strengths for the word pairs were 0.0269.

### Experimental design and procedure

In the vowel counting task, participants viewed a series of random strings of letters ranging from 3 to 8 characters each. Each string was randomly generated for each participant. Participants counted the number of vowels and typed their answer. Trials lasted four seconds.

In the cued-recall task, participants first studied 20 word pairs, one at a time, for 6 s each. Immediately afterward, they were tested on each pair, with one word presented as cue for retrieval of the target word. For each cue, participants were given six seconds to type their answers into a textbox. For example, if participants learned “brain-tape” in the study phase, in the test phase they would be presented with “brain-???,” and would attempt to retrieve the target “tape”.

In Experiment 1, participants performed an FTT in which they repeatedly typed a sequence, 4–1–3–2–4, as quickly and accurately as possible with their non-dominant left hand (Fig. 1). They were required to type this sequence correctly 15 times per trial. There were 17 training trials and two test trials, and a 10 s break between each trial. Participants performed the same FTT task on both the training and test trials. Between the training and test trials, they performed the intervening task, either vowel counting or cued-recall, for 4 min. Before training, participants were given 10 s to complete a single warmup sequence until performed correctly. Before the test, participants were given 2 s to complete a single warmup sequence until performed correctly.

In Experiment 2, the FTT was the same as in Experiment 1. In the cued-recall group, we increased the number of word pairs from 20 to 70, drawn again from the pairs used in Gupta et al.^15^. The mean forward strength for these word pairs is 0.027. The presentation time per item remained the same, totaling 14 min. In the vowel group we increased the number of character strings from 20 to 70. Participants saw each string twice, once in block A and once in block B with the block order individually randomized. All other aspects of the intervening tasks were identical to Experiment 1.

In Experiment 3, participants completed five sequences per trial with 30 s breaks, a design known to accrue minimal RI^15^. Further, we moved the intervening task to be after the first three trials (15 sequences). Based on previous data collected in our lab^14,15^, this is a likely point where declarative learning is still taking place. Participants also performed three test trials and 31 post-test trials. We used the same vowel counting and cued-recall tasks as in Experiment 2.

## Supplementary information

Supplementary 093025

## Data Availability

All data are available at https://osf.io/yfreh/. Further information and requests for resources should be directed to and will be fulfilled by the corresponding author, TCR (trickard@ucsd.edu).

## Code availability

All stimuli, and analyses are available at https://osf.io/yfreh/. Further information and requests for resources should be directed to and will be fulfilled by the corresponding author, TCR (trickard@ucsd.edu).
