# Supplementary material for: The effects of declarative learning on early and late motor skill learning
Source: NPJ Sci Learn. 2025 Dec 27;11:5. doi: 10.1038/s41539-025-00386-3 (PMC12789694; doi:10.1038/s41539-025-00386-3)
Supplement: Supplementary file 1 — Supplementary 093025 [file 41539_2025_386_MOESM1_ESM.pdf]

Supplementary

Cue	Target	Forward Strength
algae	mold	0.014
angel	wings	0.041
ape	mammal	0.021
author	poet	0.028
barn	stable	0.016
block	street	0.04
bone	skin	0.02
cement	brick	0.032
child	mother	0.03
coffee	morning	0.025
curve	shape	0.018
elf	fairy	0.013
factory	product	0.02
frame	window	0.014
game	board	0.034
gate	latch	0.013
guard	prison	0.024
hole	circle	0.016
juice	fruit	0.035
knife	spoon	0.051
leak	spill	0.034
mask	crook	0.012
milk	cookie	0.036
native	foreign	0.056
neck	collar	0.036
object	symbol	0.014
oil	motor	0.014
paint	picture	0.036
pencil	point	0.021
quarter	dollar	0.061
report	weather	0.015
slave	worker	0.069

Cue	Target	Forward Strength
sound	speaker	0.024
stick	branch	0.067
suit	armor	0.014
tack	staple	0.024
taste	touch	0.016
throat	voice	0.039
vehicle	truck	0.013
yard	field	0.016
alley	lane	0.021
angle	corner	0.02
attic	cellar	0.04
bar	grill	0.034
beach	blanket	0.012
boat	ski	0.016
cabin	camp	0.02
chain	fence	0.022
cloth	table	0.012
college	student	0.035
dew	damp	0.014
engine	machine	0.033
fire	hose	0.036
gallon	half	0.014
garden	weed	0.026
group	meeting	0.027
head	face	0.062
jelly	grape	0.032
jury	panel	0.021
lab	science	0.029
lunch	supper	0.019
master	owner	0.01
nation	state	0.042

Cue	Target	Forward Strength
nature	trail	0.023
novel	story	0.034
office	doctor	0.014
opera	tenor	0.021
pants	clothes	0.033
people	world	0.014
range	rifle	0.015

Supplementary Table 1. Cues were associated with target words. The forward strength is a measure of how closely related the cue word is to the target where the high targets have higher forward strengths.
